# Exercise-induced myokines and the muscle–tumor axis in obesity-driven gynecological cancers: a narrative review

**DOI:** 10.3389/fonc.2026.1896302

**Published:** 2026-08-11

**Authors:** Ersilia Nigro, Greta Palmieri, Marta Mallardo, Attilio Di Spiezio Sardo, Aurora Daniele, Alessandra Gallo

**Affiliations:** 1 Dipartimento di Scienze e Tecnologie Ambientali Biologiche e Farmaceutiche (DISTABiF), University of Campania Luigi Vanvitelli, Caserta, Italy; 2 Dipartimento di Medicina Molecolare e Biotecnologie Mediche, Universita degli Studi di Napoli Federico II, Naples, Italy; 3 Universita Telematica Pegaso, Naples, Italy; 4 Dipartimento di Sanita' Pubblica, Azienda Ospedaliera Universitaria Federico II, Naples, Italy

**Keywords:** cervical cancer, endometrial cancer, exercise, exerkines, immune modulation, metabolic dysfunction, myokines, obesity

## Abstract

Obesity represents a cornerstone risk factor for gynecological malignancies, particularly endometrial carcinoma and specific ovarian cancer subtypes. This association is driven by a systemic milieu of chronic low-grade inflammation, adipokine dysregulation, and hyperinsulinemia. Conversely, skeletal muscle is now recognized as a dynamic endocrine organ that, through contraction-induced secretion of myokines, orchestrates a “muscle–tumor axis” capable of antagonizing pro-tumorigenic signaling. This narrative review aims to synthesize current evidence regarding the role of exercise-induced myokines in obesity-driven gynecological cancers, elucidating the mechanisms through which these molecules reshape the systemic and tumor microenvironment. A comprehensive search of PubMed/MEDLINE, Scopus, and Web of Science was conducted up to early 2026, focusing on myokine signaling, exercise physiology, and gynecological oncology. Chronic obesity significantly impairs the skeletal muscle secretory profile, fostering an environment conducive to tumor progression. Exercise-induced secretion of key myokines, specifically IL-15, Irisin (FNDC5), SPARC, and FGF21, counteracts these effects through multiple mechanisms. Irisin and SPARC directly inhibit cell proliferation and epithelial-mesenchymal transition (EMT) by modulating the PI3K/Akt/mTOR pathway. IL-15 enhances anti-tumor immune surveillance by promoting the recruitment and cytotoxic activity of Natural Killer (NK) cells. Furthermore, FGF21 and Irisin facilitate metabolic reprogramming by inducing adipose tissue browning and restoring the insulin/IGF-1 axis homeostasis. Myokines may serve as critical biological mediators linking physical activity to cancer protection. Understanding these exercise-induced molecular transducers may provide a robust rationale for developing “precision exercise” protocols as a tailored non-pharmacological strategy to improve oncological outcomes in gynecological cancer patients.

## 1 Introduction

Obesity has emerged as a major global health challenge and represents a well-established risk factor for several gynecological malignancies, particularly endometrial cancer and selected subtypes of ovarian cancer (1). Although cervical cancer is etiologically driven by persistent high-risk Human Papillomavirus (HPV) infection rather than metabolic dysfunction alone, emerging evidence underscores that obesity-associated systemic low-grade inflammation, altered adipokine profiles, and hyperinsulinemia can impair host immune surveillance, thereby acting as critical co-factors that accelerate cervical carcinogenesis and worsen prognosis (2). Consequently, exploring the muscle-tumor axis provides valuable insights across the full spectrum of gynecological malignancies.

The link between excess adiposity and tumor development is mediated by a complex interplay of metabolic, hormonal, and inflammatory alterations that collectively create a permissive environment for carcinogenesis (3). While these mechanisms have been extensively investigated, increasing attention is now being directed toward the role of systemic metabolic regulation and inter-organ communication in shaping cancer risk and progression (4). In this context, skeletal muscle has recently gained recognition as a key regulator of whole-body homeostasis through its endocrine function, mediated by the release of myokines. These muscle-derived molecules influence metabolic balance, immune responses, and inflammatory signaling, suggesting a potential role in modulating tumor biology (5). Physical activity, long associated with reduced cancer risk and improved outcomes, may exert its protective effects in part through the modulation of skeletal muscle endocrine function (6, 7). However, the molecular mechanisms underlying this relationship remain incompletely understood, particularly in the setting of obesity-associated gynecological cancers. The concept of a muscle-tumor axis provides a novel framework to interpret these interactions, highlighting how alterations in muscle function and myokine secretion are hypothesized to contribute to a tumor-promoting or tumor-suppressive systemic environment (8). However, it must be emphasized that current evidence supporting this axis is predominantly derived from preclinical models, and robust causal validation demonstrating that muscle-derived factors directly reshape the clinical oncogenic landscape in humans remains limited (9, 10).

**Figure 1:**
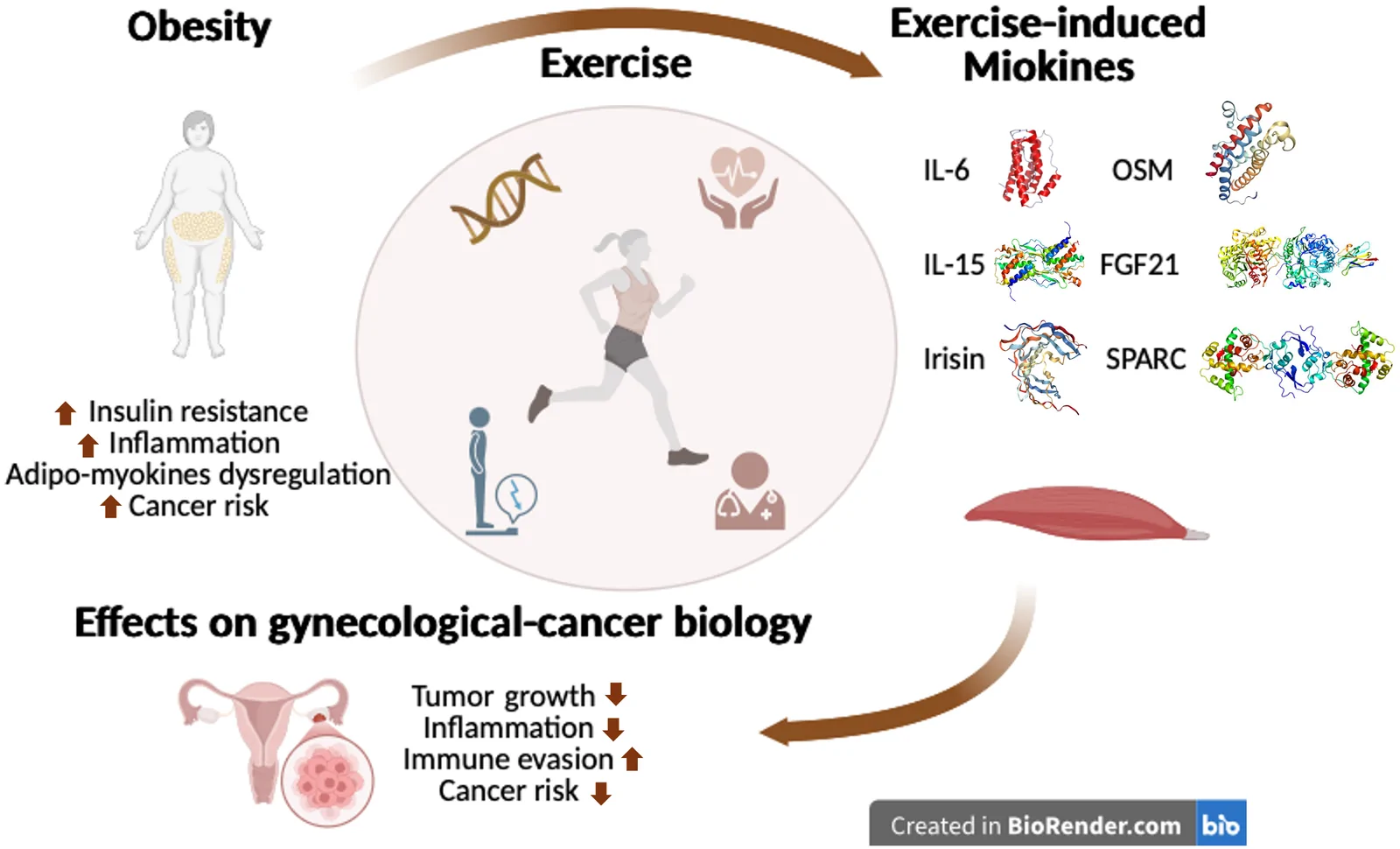
The muscle–tumor axis as a molecular modulator of obesity-associated gynecological cancers through exercise–induced myokines. Schematic overview of the systemic and local protective effects of physical exercise. Skeletal muscle contraction stimulates the synthesis and release of specific myokines (such as IL-6, IL-15, Irisin, SPARC, and FGF21). These signaling molecules enter the circulation and directly counteract the pro-tumorigenic alterations typically driven by chronic obesity (e.g., insulin resistance, hyperinsulinemia, low-grade chronic inflammation, and adipokine dysregulation), ultimately inhibiting tumor growth, angiogenesis, and metastatic potential in endometrial and ovarian cancer microenvironments. (Created with BioRender.com).

In this narrative review, we aimed to synthesize current evidence on the role of exercise-induced myokines in obesity-related gynecological cancers, with a focus on their mechanistic relevance and potential translational implications. By integrating metabolic, endocrine, and oncological perspectives, this work seeks to provide a comprehensive overview of the emerging interplay between skeletal muscle and tumor biology and to identify potential avenues for future research and therapeutic intervention. A general overview of the molecular crosstalk and systemic pathways involved in this muscle–tumor axis is schematically depicted in **Figure 1**.

## 2 Methodology

This narrative review was conducted to synthesize current evidence regarding the role of exercise-induced myokines in obesity-associated gynecological cancers and to explore their potential mechanistic and translational relevance. A comprehensive literature search was performed using the electronic databases PubMed/MEDLINE, Scopus, and Web of Science from database inception through January and May 2026. Additional articles were identified through manual screening of reference lists from relevant reviews and original research papers. The search strategy combined controlled vocabulary and free-text terms related to skeletal muscle signaling, exercise biology, obesity, and gynecological malignancies. Representative keywords included: “myokines,” “exerkines,” “skeletal muscle,” “exercise,” “physical activity,” “obesity,” “metabolic dysfunction,” “endometrial cancer,” “ovarian cancer,” “cervical cancer,” “tumor microenvironment,” and “immune modulation”.

To ensure transparency, reproducibility, and methodological rigor, the following specific selection criteria were established and applied during the literature screening process:

Inclusion Criteria: 1. Peer-reviewed original research articles (including preclinical *in vitro* and *in vivo* models, translational research, observational cohorts, and randomized clinical trials) and high-quality scientific reviews. 2. Studies directly investigating the mechanistic, physiological, or clinical role of muscle-derived factors (myokines) within the context of cancer biology, systemic metabolic regulation, or exercise oncology. 3. Target malignancies strictly restricted to gynecological cancers (endometrial, ovarian, and cervical cancers). 4. Full-text publications available in the English language.

Exclusion Criteria: 1. Conference abstracts, case reports, editorials, technical notes, and doctoral dissertations. 2. Studies focusing strictly on non-gynecological cancer sites without systemic relevance to the muscle-tumor axis framework. 3. Articles exploring general physical activity or dietary changes without analyzing skeletal muscle endocrine functions or the myokine secretome.

Given the narrative nature of this review, formal quality scoring and meta-analytic techniques were not applied. Instead, emphasis was placed on integrating mechanistic insights with emerging clinical data to provide a comprehensive and hypothesis-generating overview of this evolving field.

## 3 Obesity and gynecological cancers: epidemiological and biological interplay

### 3.1 Epidemiological landscape

The global surge in obesity prevalence directly correlates with the rising incidence of gynecological malignancies, particularly among reproductive-age women (8–11). Endometrial cancer (EC) exhibits the most profound association, with obesity accounting for approximately 40-50% of its incidence (8). This epidemiological link extends to specific ovarian cancer subtypes, including low-grade serous and endometrioid carcinomas, suggesting a shared metabolic vulnerability (8). Beyond primary risk, obesity serves as a critical determinant of clinical management; the presence of insulin resistance and cardiovascular comorbidities complicates surgical and systemic therapeutic planning, often limiting fertility-sparing options in premenopausal patients and worsening overall survival across all stages.

### 3.2 Pathophysiological mechanisms of adipose-driven carcinogenesis

Obesity facilitates a permissive oncogenic landscape through a synergistic convergence of endocrine, metabolic, and inflammatory aberrations (12). In the state of chronic energy excess, adipose tissue undergoes pathological remodeling, transitioning from a homeostatic reservoir to a dysfunctional endocrine organ (13). Hypertrophic adipocytes and infiltrating macrophages orchestrate a systemic “pro-tumorigenic milieu” through the following key axes: a) The Steroid-Hormone Axis: Peripheral adipose tissue serves as the primary site for the aromatization of androgens into estrogens. In obese patients, the resulting chronic hyperestrogenism, unpacked by progesterone, drives sustained endometrial epithelial proliferation and genomic instability, elevating the risk of malignant transformation (11); b) Insulin/IGF-1 Hyperactivation: Systemic insulin resistance leads to compensatory hyperinsulinemia, which suppresses the hepatic synthesis of Sex Hormone-Binding Globulin (SHBG) and IGF-Binding Proteins (IGFBPs) (11). This increases the bioavailability of free estrogens and Insulin-like Growth Factor-1 (IGF-1). IGF-1, acting as a potent mitogen, triggers the PI3K/Akt/mTOR and Ras/MAPK pathways, favoring anabolic reprogramming and anti-apoptotic signaling (14). Crucially, this severe dysregulation of key adipokines (such as adiponectin, leptin, and visfatin) and the underlying insulin resistance represent a shared oncometabolic bridge found in other systemic disorders, including gestational diabetes mellitus (GDM), where altered adipokine profiles serve as critical prognostic indicators of metabolic dysfunction (15); c) Chronic Low-Grade Inflammation: Dysfunctional adipocytes secrete a distinct profile of pro-inflammatory adipokines and cytokines (e.g., TNF-α, IL-6, and Leptin). This chronic inflammatory state activates the JAK/STAT and NF-κB transcriptional programs, which exacerbate oxidative stress, induce DNA damage, and promote an immunosuppressive microenvironment that facilitates tumor evasion (12); d) Hypoxia and Lipotoxicity: Rapid adipose expansion lebad to localized hypoxia, stabilizing Hypoxia-Inducible Factors (HIFs) that drive angiogenesis and metabolic adaptation (the Warburg effect). Concomitantly, lipid overflow and mitochondrial dysfunction generate reactive oxygen species (ROS), further increasing the mutational burden within the epithelial niche (13).

## 4 The biological paradigm of exercise in cancer: beyond weight loss

### 4.1 Systemic adaptations and redox homeostasis

Regular physical activity is no longer viewed merely as a behavioral modifier but as a systemic biological intervention. Beyond its established role in reducing all-cause mortality and metabolic disorders, exercise orchestrates a multi-layered defense against malignancy by restoring hormonal balance and modulating immune function (16). A pivotal, yet often overlooked, mechanism is the fine-tuning of redox homeostasis. While acute exercise-induced surges in reactive oxygen species (ROS) act as transient signaling molecules, chronic training triggers a superior antioxidant counter-response (17). By upregulating DNA repair systems and antioxidant enzymes, exercise effectively mitigates the oxidative damage and mutagenic potential typical of the obesity-driven endometrial environment (18).

### 4.2 Immunomodulation and the “cold-to-hot” tumor transition

Exercise exerts profound immunomodulatory effects that are particularly relevant in gynecological oncology, where the tumor microenvironment is often immunosuppressive (19).

Physical activity facilitates the mobilization and infiltration of Natural Killer (NK) cells and cytotoxic CD8+ T-cells, while simultaneously promoting macrophage polarization toward anti-tumor (M1) phenotypes (20). This heightened immune surveillance may provide a biological rationale for integrating supervised multimodal exercise, including aerobic and resistance training, as a synergistic complement to emerging immunotherapies, potentially improving survival outcomes in ovarian and cervical cancers (20).

### 4.3 The muscle–tumor axis: skeletal muscle as an endocrine driver

The most significant shift in exercise oncology is the redefinition of skeletal muscle from a contractile tissue to one of the body’s largest endocrine organs (21). Indeed, in response to mechanical stimuli, myocytes release a sophisticated repertoire of peptides, metabolites, and extracellular vesicles, collectively termed myokines. This secretory capacity establishes a complex inter-organ communication network that has been shown in preclinical models to modulate pro-tumorigenic pathways (22).

Crucially, in obese patients, this axis is often compromised by myosteatosis, the lipid infiltration of muscle tissue, which leads to “myokine silencing” and impaired oxidative capacity (23). This dysfunctional state exacerbates the pro-inflammatory milieu of adipose tissue, creating a vicious cycle of metabolic derangement. Structured exercise may act as a potent countermeasure by remodeling the muscle secretome and restoring physiological endocrine equilibrium. Remarkably, these protective adaptations occur independently of substantial weight loss, suggesting that the muscle-derived “oncosuppressive pulses” are sufficient to attenuate mitogenic signaling and enhance therapeutic responsiveness even in the presence of persistent adiposity (16). This concept aligns with clinical evidence showing that long-term structured training programs significantly modulate and improve circulating adiponectin and metabolic profiles in obese individuals in relation to physical performance, regardless of massive changes in overall body fat mass (15, 24).

### 4.4 Physiological and molecular dimensions of myokine-tumor microenvironment crosstalk

To fully appreciate the translational potential of the muscle-tumor axis, the underlying molecular and physiological paradigms governing the crosstalk between contracting skeletal muscle and the tumor microenvironment (TME) must be explicitly established. Myokines do not merely exert broad systemic metabolic downstream effects; instead, they function as targeted signaling macromolecules that directly penetrate and reshape the immunosuppressive oncogenic niche through distinct mechanistic routes. At the cellular level, this bidirectional communication is heavily characterized by immune cell reprogramming within the TME. Under sedentary and obese conditions, the gynecological tumor niche is typically infiltrated by pro-tumorigenic M2-like tumor-associated macrophages (TAMs) and myeloid-derived suppressor cells (MDSCs) that shield cancer cells from host defense. Conversely, the pulsatile secretion of specific myokines during skeletal muscle contraction disrupts this protective barrier. These muscle-derived factors physically enhance the chemotaxis, trans-endothelial migration, and ultimate homing of cytotoxic CD8+ T-lymphocytes and Natural Killer (NK) cells directly into the core of the tumor, effectively restoring host-mediated immunosurveillance.

Beyond immunomodulation, myokines directly engage with transmembrane receptors on cancer cells to suppress cell-autonomous survival circuits. The primary physiological pathways targeted include the phosphoinositide 3-kinase/protein kinase B/mammalian target of rapamycin (PI3K/Akt/mTOR) axis—which is characteristically hyperactivated in obesity-driven endometrial and ovarian cancers—as well as the Janus kinase/signal transducer and activator of transcription 3 (JAK/STAT3) cascade. By inducing receptor-mediated intracellular blocks or upregulating endogenous inhibitors, myokines can force cell cycle arrest and trigger apoptosis in malignant lineages. Furthermore, the muscle endocrine secretome actively interferes with paracrine TME components by suppressing vascular endothelial growth factor (VEGF)-driven angiogenesis and limiting the matrix metalloproteinase (MMP) adaptations required for epithelial-to-mesenchymal transition (EMT) and distal metastasis. By anchoring the review within these foundational molecular networks, the subsequent analysis of individual myokine variations and clinical trial frameworks can be interpreted through a unified, mechanically rigorous lens.

## 5 Key myokines in gynecological cancer modulation

### 5.1 A context-dependent framework of myokine pleiotropy

To understand the biological impact of the muscle-tumor axis in gynecological malignancies, it is critical to move away from binary classifications of myokines as strictly tumor-suppressive or tumor-promoting. As key signaling mediators, myokines exhibit profound pleiotropy, a phenomenon inherently context-dependent, where the final biological outcome is dictated by receptor expression profiles, downstream intracellular cascades, and the localized cellular microenvironment (25–27).

Taking into account such context-dependent actions of myokines liter, we propose a unifying framework structured around three distinct contextual axes:

- Kinetics of Exposure and Exercise Modalities (Acute vs. Chronic): Exercise-induced muscle contractions operate as an endogenous, pulsatile stressor, generating acute and transient spikes of circulating myokines. The physiological magnitude, temporal kinetic profile, and molecular composition of this contracting skeletal muscle secretome are strictly dictated by specific exercise modalities, intensities, and cumulative volumetric doses. Acute, high-intensity aerobic training (e.g., cycling or running at >75% VO2max for 30–45minutes) induces massive metabolic stress, characterized by accelerated glycogen depletion and transient intracellular hypoxia within Type I and Type II myofibers. This phenomenon triggers the rapid up-regulation of some myokines, such as interleukin-6 (IL-6) and PGC-1α-driven Irisin (28, 29). Conversely, progressive resistance training (e.g., 3 sets of 8–12 repetitions at 70–80% of 1-Repetition Maximum, performed 3 times weekly) introduces a distinct mechanical tension and microstructural myofibrillar strain. This mechanical loading activates the secretome toward localized hypertrophic, regenerative, and immunomodulatory factors. This structural strain preferentially drives the expression and autocrine/paracrine release of Secreted Protein Acidic and Rich in Cysteine (SPARC) and Interleukin-15 (IL-15) (30, 31). To exploit these complementary pathways, combined concurrent training frameworks (aerobic plus resistance modalities) have emerged as a highly effective clinical architecture in exercise oncology. Concurrent training simultaneously stimulates the transcriptional networks of both metabolic and mechanical cascades, maximizing the aggregate breadth of the muscle endocrine response (32).
- Receptor Dynamics and Signaling Subtypes: The cellular response is strictly governed by the spatial configuration of receptor complexes. In healthy physiology, myokines engage in classical “cis-signaling” by binding directly to membrane-bound receptors (e.g., IL-6R or OSMR) co-expressed with the gp130 subunit, driving homeostatic, anti-inflammatory cascades (28, 79). In the obesity-associated tumor microenvironment (TME), however, metalloproteinases (ADAM10/17) shed these membrane receptors, generating soluble variants (sIL-6R/sOSMR). Because gp130 is ubiquitously expressed while membrane receptors are highly restricted, these hyper-stable soluble complexes bind to and hyperactivate cells normally unresponsive to the isolated myokine, switching the downstream JAK/STAT3 and MAPK pathways from homeostatic checkpoints to aggressive oncogenic drivers of survival and chemoresistance (33, 80).
- Tumor Subtype and Microenvironmental Crosstalk: Host tissues, cellular lineages, and distinct gynecological subtypes exhibit highly variable receptor landscapes and epigenetic signatures. This heterogeneity dictates that a single myokine can trigger opposing biological outcomes across different niches, highlighting the necessity of mapping complex physical and systemic barriers to understand target tissue vulnerability (25). Furthermore, the local TME functions as an integrated system where hypoxia and chronic adiposity alter cellular responses (26). This microenvironmental dependency is profoundly evident in the paradoxical behavior of key cytokines and matricellular factors. For example, while acute muscle-derived IL-6 or Irisin exposure effectively blocks the hyperactivated PI3K/Akt/mTOR survival pathway in endometrioid endometrial carcinomas, the identical IL-6 signal is hijacked via trans-signaling in advanced serous ovarian cancer to hyperactivate the JAK/STAT3 cascade (33). Similarly, the behavior of the oncosuppressive secretome is heavily dictated by localized crosstalk: the acute release of Oncostatin M (OSM) induces growth arrest in homeostatic epithelial cells, yet within a TGF-β-rich and hypoxic ovarian or cervical TME, chronically elevated OSM or tissue-specific SPARC upregulation are subverted by cancer-associated fibroblasts and tumor-associated macrophages to drive angiogenesis, matrix metalloproteinase production, and epithelial-to-mesenchymal transition (34–36). Furthermore, metabolic regulators like FGF21 can act as homeostatic rejuvenators systemically, but their localized tissue variations are easily altered by therapeutic stressors within specific ovarian niches, transforming a muscle-derived adaptation into an active mechanism to promote tumor persistence (27). By applying this context-dependent model, the paradoxical behaviors of key myokines can be systematically deconstructed and reconciled.

Physical activity, through the release of myokines, exerts a pivotal role in cancer (37–39) and in particular in endometrial cancer (40). Here, we focus on the existing literature concerning the main myokines involved in oncology, taking into account the above-mentioned context of action.

### 5.2 Interleukin-6

Although traditionally viewed as a pleiotropic, pro-inflammatory cytokine that drives tumor progression when chronically elevated, IL-6 exhibits distinct biological behavior when released transiently by contracting skeletal muscle during exercise (28).

In the context of obesity-associated gynecological malignancies, chronic systemic low-grade inflammation persistently elevates circulating IL-6 derived from dysfunctional adipose tissue or the tumor niche itself (41). This chronic exposure activates trans-signaling pathways via the JAK/STAT3 axis, promoting tumor cell survival, migration, and chemoresistance in epithelial ovarian and endometrial carcinomas, and correlating with aggressive features such as uterine serous papillary histology (33).

Conversely, acute exercise-induced pulsatile spikes of muscle-derived IL-6 act as an anti-inflammatory and metabolic sensor. This transient myokine pulse stimulates glucose uptake, increases systemic insulin sensitivity, and coordinates anti-inflammatory loops primarily by inducing interleukin-10 (IL-10) and interleukin-1 receptor antagonist (IL-1Ra). In parallel, regular training has been shown to actively downregulate baseline IL-6, highlighting its potential to disrupt chronic oncogenic inflammatory signaling if harnessed via tailored exercise protocols (29).

### 5.3 Oncostatin M

While acutely released during physical exercise, chronic elevation of OSM in the gynecological tumor microenvironment acts as a potent pro-tumorigenic driver, showing how chronic exposure subverts an otherwise physiological signal.

OSM is secreted by contracting muscle and exerts anti-proliferative effects across multiple epithelial cancer models (42). By modulating STAT3 signaling, OSM can induce growth arrest and apoptosis, supporting a tumor-suppressive role when produced within the physiological context of exercise (43). In the context of gynecological malignancies, preclinical studies indicate that the OSM, OSMR, STAT3 axis may contribute to tumor progression, angiogenesis, invasion, and therapy response. Both OSM and its receptors are commonly co-expressed in malignant ovarian epithelial cells, suggesting a potential autocrine signaling loop (44). In *in vitro* studies, Recombinant OSM promotes proliferation of SKOV3 ovarian cancer cells via STAT3, ERK1/2, and p38 pathways (45). In support of such data, single-cell analyses showed that the OSM receptor is highly expressed in ovarian cancer cells, while the ligand is produced by tumor-associated macrophages and promotes proliferation and metastasis. Antibodies targeting OSMR blocked signaling and inhibited tumor growth *in vitro* and *in vivo*, providing proof-of-concept for immunotherapy (46). OSM expression is significantly higher in endometrial cancers than in normal tissues and correlates with tumor stage, grade, myometrial invasion, and lymph node metastasis (47). Experimental *in vitro* models demonstrate that OSM promotes angiogenesis, migration, and invasion via STAT3 activation (34). *In vivo*, OSM overexpression enhanced tumor growth in nude mice, supporting its role as a tumor promoter (48). In cervical cancer, only few data are available; the oncostatin M receptor (OSMR) is commonly copy number gained and overexpressed in advanced cervical SCC, changes that are associated with significantly worse clinical outcomes (35, 48). OSMR overexpression in cervical SCC cells results in enhanced responsiveness to OSM, which induces several pro-malignant effects, including a pro-angiogenic phenotype and increased cell migration and invasiveness (35). *In vitro* OSM pretreatment can sensitize cervical cancer cells to cisplatin-based chemoradiotherapy through STAT3 signaling (49). OSM is now recognized as an exercise-responsive myokine involved in immune cell recruitment/phenotype in skeletal muscle following aerobic exercise. There is also indication that OSM can rise after acute exercise in experimental models, supporting its inclusion among exercise-linked myokines (49). Although OSM is characterized as an exercise-responsive myokine with antiproliferative potential in generic epithelial models, its role in gynecologic oncology appears predominantly pro-tumorigenic, promoting angiogenesis and the epithelial-mesenchymal transition (EMT) via the STAT3 signaling pathway. While it is released acutely during physical exercise, its chronic elevation within the gynecologic tumor microenvironment acts as a potent pro-tumorigenic factor, demonstrating how prolonged exposure alters a signal that is otherwise physiological. These findings suggest that it may be a crucial myokine in gynecologic cancers, although its specific involvement has yet to be fully elucidated.

### 5.4 SPARC

The matricellular protein SPARC is upregulated following endurance exercise and has been shown to promote caspase-mediated apoptosis while inhibiting cell-cycle progression (30). Experimental models suggest that SPARC contributes significantly to the anti-tumor effects of physical activity, particularly through its influence on extracellular matrix remodeling. Indeed, SPARC is increasingly viewed as a matricellular regulator of the tumor microenvironment rather than a classical cytokine (50). Its expression is inversely correlated with malignancy, and reduced expression appears important for ovarian carcinogenesis (51). Exogenous SPARC inhibits proliferation and induces apoptosis specifically in cancer cells, indicating a direct *in vitro* antitumor effect (52). Interestingly, SPARC significantly inhibits integrin-mediated adhesion and suppresses AKT and MAPK survival pathways in ovarian cancer cells (53). In endometrial cancer, SPARC regulates extracellular matrix interactions and can modulate adhesion, migration, invasion, apoptosis, and epithelial-to-mesenchymal transition (EMT) (54). Overexpression of SPARC together with fibronectin activates fibroblasts, enhancing cancer cell mobility and invasion (55). SPARC is significantly upregulated in cervical squamous cell carcinoma and correlates with poorer prognosis (56). Functional studies show it promotes proliferation, invasion, migration, colony formation, and EMT activation (36). Although demonstrated strongly in colon tumorigenesis models, SPARC can be suggested as an exercise-induced myokine and showed it can mediate anti-tumor effects (30). However, although SPARC exerts clear tumor-suppressive effects in ovarian carcinoma by inducing apoptosis, it is associated with a poorer prognosis and increased invasiveness in squamous cell carcinomas of the cervix and endometrium; thus, in a context-dependent manner, SPARC induces apoptosis in certain ovarian carcinoma subtypes via stress-activated pathways, yet promotes epithelial-mesenchymal transition (EMT) and invasion when chronically overexpressed in a TGF-β-rich microenvironment. These discrepancies suggest that the therapeutic efficacy of exercise-induced myokines is heavily dictated by the unique epigenetic and receptor landscape of the target gynecological tissue. The divergent roles of OSM and SPARC highlight the need for further mechanistic studies to refine precision exercise protocols.

### 5.5 Interleukin-15

IL-15 plays a dual role in muscle hypertrophy and immune activation. By enhancing natural killer and CD8+ T-cell activity, IL-15 may strengthen anti-tumor immunity (57). Impaired IL-15 signaling observed in obesity further underscores the importance of muscle health in cancer defense (30). IL-15 is expressed in ovarian tumor tissues and ascites, but levels are often insufficient to sustain effective immune responses. Reduced IL-15 signaling is associated with impaired NK-cell infiltration and function within the tumor microenvironment. Preclinical models show that IL-15 enhances NK-cell–mediated cytotoxicity against ovarian cancer cells, promotes CD8^+^ T-cell persistence, inhibits tumor growth indirectly via immune activation (57). Local IL-15 transpresentation in ovarian cancer cells resulted in superior NK-cell proliferation, cytotoxicity, granzyme-B secretion, and tumor killing in spheroid models (58). In endometrial cancer, data are more limited, but IL-15 expression has been detected in normal endometrium as well as in endometrial tumors, often at reduced levels, thus IL-15 contributing to weak NK-cell surveillance, immune escape in obesity-associated endometrial cancer (30). Regarding cervical cancer, IL-15 has been studied mainly in the context of HPV infection, antiviral and antitumor immunity. Higher IL-15 expression correlates with increased NK-cell and CD8^+^ T-cell infiltration, better immune control of HPV-associated lesions. Lastly, experimental studies suggest that IL-15 enhances cytotoxic lymphocyte activity, improving responses to immunotherapy and radiotherapy. Systematic reviews support that acute exercise increases circulating IL-15 (31, 57). A gynecology-focused epidemiology paper also explicitly lists IL-15 among exercise-induced myokines relevant to gynecological cancer protection (21).

### 5.6 Fibroblast growth factor 21

Although primarily classified as a hepatokine, FGF21 is also released from skeletal muscle during metabolic stress. It acts as a master regulator of systemic metabolic health. Released by skeletal muscle during metabolic adaptations to endurance training, FGF21 stimulates adipose tissue browning and improves insulin sensitivity, potentially reversing the systemic hormonal derangements that drive endometrial carcinogenesis (57). However, its profile remains dualistic, as certain preclinical data suggest that localized FGF21 upregulation might paradoxically support tumor survival or modulate cisplatin resistance within specific ovarian cancer niches (57). By integrating these mediators into a comparative synthesis, it becomes clear that the skeletal muscle endocrine response is a coordinated network where IL-6 balances acute energy mobilization against chronic inflammation, IL-15 drives targeted immunosurveillance, and FGF21 acts as a metabolic rejuvenator. Nevertheless, a substantial translational imbalance persists; while preclinical mechanisms for IL-15 and FGF21 are promising, robust clinical interventional trials validating their isolated causal contributions to human tumor outcomes remain under-investigated compared to classical inflammatory pathways.

It improves insulin sensitivity and reduces systemic inflammation, indirectly targeting pathways central to obesity-associated carcinogenesis. In endometrial cancer, strong clinical data are available, suggesting it as a diagnostic and prognostic biomarker (57). A study including 233 patients evaluated whether FGF21 could serve as a biomarker for endometrial cancer. FGF21 concentrations were significantly higher in malignant lesions, supporting diagnostic potential (59). Elevated FGF21 correlated with clinical and histopathological progression and appears useful as both a diagnostic and prognostic factor in endometrioid endometrial carcinoma (31). For the ovarian cancer, upregulated FGF21 reduced cisplatin sensitivity, decreased apoptosis, and improved growth of treated ovarian cancer cells. Conversely, FGF21 downregulation increased cisplatin sensitivity and enhanced apoptosis (60). Interestingly, FGF21 was found to be significantly decreased in ascites and plasma of aged epithelial ovarian cancer models (31). Acute exercise increases circulating FGF21 in humans/mice. Reviews/meta-analyses discuss how FGF21 often rises with aerobic/endurance exercise, with variability by sex/training status (36, 60). FGF21 may contribute to the metabolic stress adaptation related to exercise potentially acting as a pro-survival/pro-resistance molecule in certain tumor contexts (e.g., ovarian chemoresistance). FGF21 presents a dualistic profile: it acts as a master regulator of metabolic health by improving insulin sensitivity, yet clinical evidence suggests it may inadvertently support tumor survival and cisplatin resistance in specific ovarian cancer contexts. We can conclude that these findings underscore the necessity of ‘precision exercise’ timing to maximize metabolic benefits while avoiding sustained pro-survival signaling within the tumor niche.

### 5.7 Irisin

Irisin is a thermogenic myokine produced through the proteolytic cleavage of the fibronectin type III domain-containing protein 5 (FNDC5), a process primarily driven by the exercise-induced activation of the PGC-1α pathway in skeletal muscle (61). In the complex landscape of the muscle-tumor axis, irisin has emerged as a fundamental pleiotropic mediator capable of exerting both direct oncosuppressive actions and indirect systemic metabolic benefits (62, 63).

**Figure 2:**
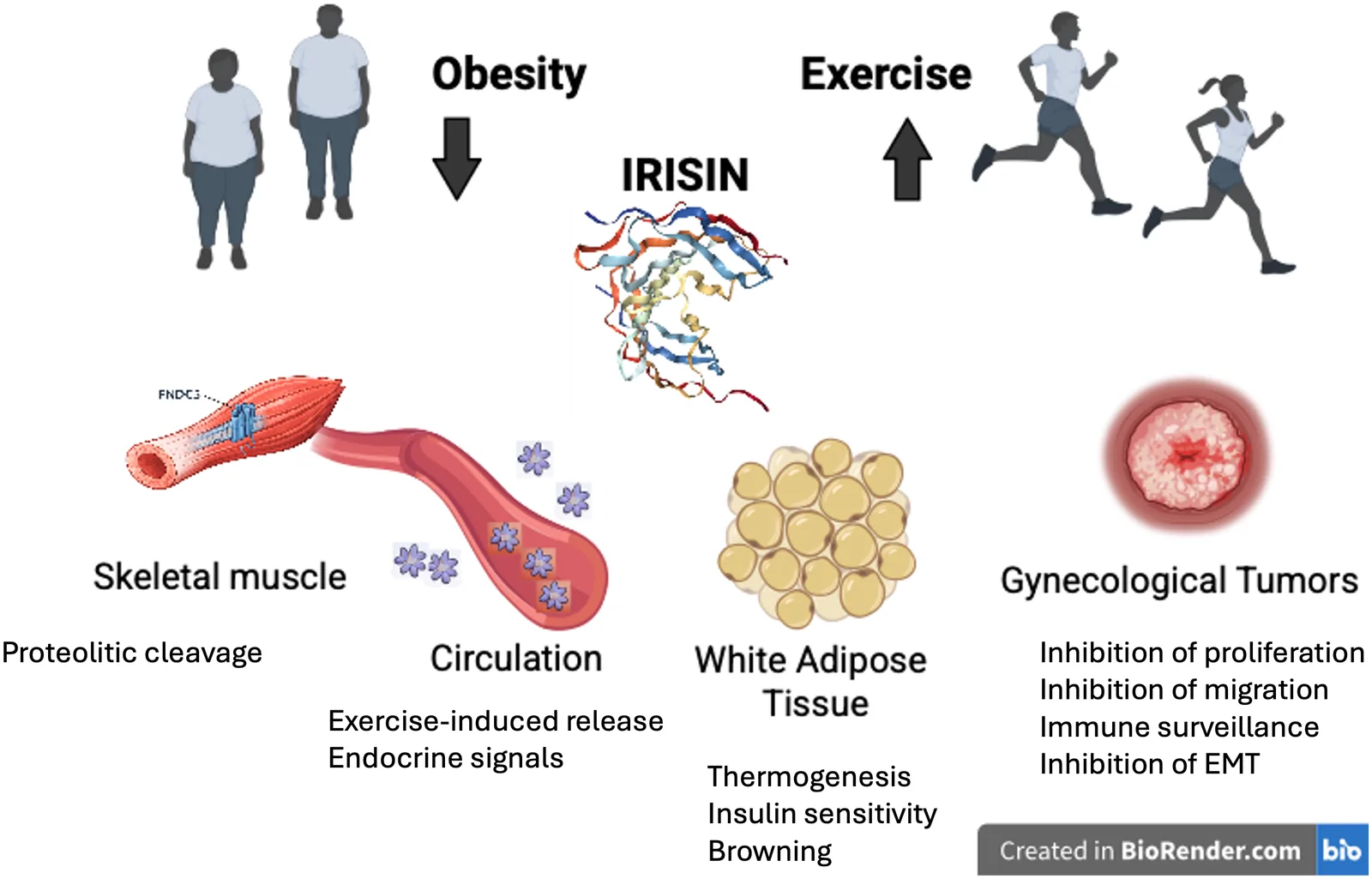
Irisin as a mediator of the muscle–tumor axis in obesity-associated gynecological cancers. Exercise stimulates skeletal muscle to release irisin through FNDC5 cleavage. Circulating irisin exerts metabolic effects, including improved insulin sensitivity, reduced inflammation, and browning of white adipose tissue. In gynecological cancers, irisin may modulate tumor biology by inhibiting proliferation, migration, and invasion while promoting apoptosis and immune surveillance. Obesity-associated impairment of irisin secretion may contribute to a tumor-permissive environment, whereas exercise-induced restoration of irisin signaling supports tumor-inhibitory adaptations. (Created with BioRender.com).

From a mechanistic perspective, irisin directly interferes with the molecular hallmarks of gynecological malignancies (64–66). Specifically, in preclinical studies, Irisin has demonstrated anti-tumorigenic effects on gynecological cancer cells by downregulating key proliferation pathways, inducing cell cycle arrest, and reversing the epithelial-mesenchymal transition (EMT) cascade. The comprehensive intracellular signaling pathways, surface receptor interactions, and downstream molecular targets modulated by exercise-induced Irisin in the tumor microenvironment are schematically illustrated in **Figure 2**. Recent evidence indicates that irisin treatment significantly downregulates the PI3K/Akt/mTOR signaling cascade, a pathway frequently overactivated in endometrial cancer, thereby inhibiting uncontrolled cell proliferation and inducing mitochondrial-mediated apoptosis (19, 67, 68).

Furthermore, irisin serves as a potent inhibitor of the epithelial–mesenchymal transition (EMT); by modulating the expression of E-cadherin and vimentin, it effectively impairs the invasive and metastatic potential of ovarian and endometrial tumor cells (69). Beyond its direct effects on tumor signaling, irisin acts as a master regulator of systemic energy homeostasis (70, 71). By promoting the “browning” of white adipose tissue and upregulating Uncoupling Protein 1 (UCP1), it enhances thermogenesis and energy expenditure (55, 59). This metabolic reprogramming is crucial in counteracting the chronic hyperinsulinemia and the dysregulated insulin/IGF-1 axis that act as primary drivers for endometrial carcinogenesis in the context of obesity. Moreover, irisin has been shown to modulate the tumor microenvironment by enhancing the recruitment and cytotoxic activity of Natural Killer (NK) cells, thereby strengthening anti-tumor immune surveillance (72, 73). Collectively, these findings position irisin as a key translational biomarker, providing a preclinical biological rationale for the potential future development of structured exercise as a “precision medicine” tool to improve oncological outcomes in obesity-related gynecological cancers. Nevertheless, translating these cellular observations into verified clinical protocols requires substantial caution, as direct human evidence confirming that exercise-induced irisin effectively alters tumor progression *in vivo* is still missing (39).

**Table 1** shows the principal exercise-Induced myokines, including their biological functions, oncologic effects.

**Table 1:** Exercise-induced myokines: biological functions, oncologic effects, and translational relevance in obesity-associated gynecological cancers.

Myokine	Primary physiological role	Effects on tumor	Level of evidence	Data in gynecological cancers	Translational potential
Irisin	Regulates energy expenditure; promotes browning of white adipose tissue	Context-dependent (predominantly anti-tumor in preclinical models)	Preclinical and Clinical	Heterogeneous circulating levels; tissue expression documented across tumor types	Candidate biomarker and potential therapeutic target
IL-6 (muscle-derived)	Acute metabolic regulator during exercise; enhances glucose uptake and lipid oxidation	Dual: anti-tumor (acute exercise) vs pro-tumor (chronic inflammation)	Strong mechanistic; limited tumor-specific clinical data	Associated with proliferation, invasion, and poor prognosis when chronically elevated	Context-dependent biomarker
IL-15	Supports muscle hypertrophy and immune activation	Predominantly anti-tumor via immune surveillance	Preclinical with emerging translational data	Enhances NK and CD8^+^ T-cell activity; limited clinical correlation	Promising immunometabolic mediator
SPARC	Regulates extracellular matrix remodeling	Context-dependent	Mostly preclinical	Implicated in apoptosis but also invasion and EMT depending on tumor context	Potential therapeutic pathway
Oncostatin M	Cytokine involved in tissue repair and inflammatory signaling	Predominantly pro-tumor in gynecologic models	Preclinical with molecular data	Linked to angiogenesis, invasion, and metastasis	Mechanistically compelling but complex
FGF21	Master regulator of metabolic homeostasis	Indirect; metabolically protective but potentially pro-survival in tumors	Clinical metabolic data; limited oncologic studies	Elevated in endometrial cancer; may influence chemoresistance in ovarian cancer	Strong metabolic target

### 5.8 Comparative mechanistic synthesis of key mediators

When evaluated as an integrated system, the skeletal muscle endocrine response does not rely on a single signaling molecule; rather, it functions as a coordinated network where distinct myokines target different oncogenic pathways. This multidimensional secretome shifts the biological landscape from generalized systemic inflammation to localized tissue remodeling and targeted immune surveillance. Within this network, each mediator possesses a unique functional signature: while muscle-derived IL-6 balances acute energy mobilization against chronic adipose-driven inflammation, IL-15 serves as a primary driver of targeted immunosurveillance by directly stimulating cytotoxic immune cells. Concomitantly, FGF21 acts as a systemic metabolic rejuvenator to reverse hormonal derangements, while matricellular factors and cytokines like SPARC and OSM modulate extracellular matrix remodeling and cellular survival in a highly context-dependent manner.

This comparative synergy is particularly evident in gynecological oncology, with a unique metabolic and cellular landscape of each specific tumor subtype:

Endometrial Cancer: In this hyperinsulinemic and estrogen-driven niche, the co-activation of PGC-1α-driven Irisin and FGF21 acts as a metabolic master switch. These myokines primarily target systemic insulin resistance, promote adipose browning, and directly induce cell cycle arrest and mitochondrial apoptosis by shutting down the overactivated PI3K/Akt/mTOR proliferation pathway.

Ovarian Cancer: In the advanced or serous carcinoma setting, the biological priority shifts toward the local TME and the suppression of ascites-driven immune evasion. Here, the dynamic interaction between muscle-derived IL-6, IL-15, and SPARC regulates extracellular matrix remodeling and cell adhesion. While muscle-derived IL-15 works to rescue local natural killer (NK) cell competence and CD8+ T-cell infiltration, molecules like OSM and SPARC exhibit a dualistic nature, potentially being hijacked by local TGF-β-rich environments to drive chemoresistance and EMT if not carefully controlled.

Cervical Cancer: Although etiologically driven by persistent high-risk HPV infection rather than primary metabolic derangement, this malignancy remains vulnerable to the muscle-tumor axis through targeted immunomodulation. The exercise-induced pulsatile release of immunometabolic mediators—specifically IL-15 and SPARC—optimizes host immune surveillance, enhancing NK cell cytotoxicity and T-lymphocyte homing into the core of the tumor.

Despite this promising preclinical synergy, a substantial translational imbalance persists within the current literature. While the cellular mechanisms backing the oncosuppressive roles of these individual myokines are increasingly mapped, robust clinical interventional trials validating their isolated, causal contributions to human tumor outcomes and survival remain highly limited compared to classical inflammatory pathways. Future research must bridge this gap by investigating how these muscle-derived transducers interact simultaneously within the clinical oncological landscape.

## 6 Clinical and translational implications

The integration of structured physical exercise into gynecological oncology represents a critical step in translating the “muscle–tumor axis” molecular framework into tangible clinical benefits. Despite the robust biological rationale and international guidelines (e.g., ASCO, NCCN), exercise implementation remains suboptimal in routine care. On the other hand, clinical relevance is supported by recent data indicating that the impact of physical activity on endometrial cancer survival may vary depending on the primary treatment (74).

In patients with gynecological malignancies, particularly endometrial cancer, adherence is often hindered by obesity-related comorbidities, treatment-induced side effects, and logistical constraints. However, emerging evidence suggests that accessible, low-impact, and home-based interventions, monitored via telehealth, can significantly enhance feasibility and long-term engagement in this population (75) also during chemotherapy with significant effects on anti-inflammatory biomarkers (76).

### 6.1 Analysis of emerging clinical trials

Translational research is currently evaluating diverse intervention windows, ranging from pre-treatment preconditioning to long-term survivorship.

- Interventions During Active Treatment: Trials such as NCT05761561 investigate the synergy between supervised exercise and nutritional support during chemotherapy to improve treatment tolerance and quality of life (QoL).
- Exercise Preconditioning: The NCT05029154 trial explores high-intensity interval training (HIIT) prior to surgery, aiming to optimize cardiorespiratory fitness and improve surgical outcomes through the acute mobilization of oncosuppressive myokines.
- Digital Health and Remote Monitoring: Innovative approaches, including mobile apps (NCT05743517) and personalized online programs (NCT06709534), are being deployed to overcome geographical barriers while assessing novel biomarkers such as microbiome composition and systemic inflammatory profiles.
- Survivorship and Functional Recovery: Programs like STRIVE Cardio (NCT06534008) and the NCT06786091 rehabilitation study focus on cardiovascular health and the management of late treatment effects, including pelvic and sexual dysfunction.

Several ongoing clinical trials are currently evaluating the impact of structured physical activity in gynecological cancer patients. While the majority of historical data focus on endometrial cancer survivors, attention is rapidly shifting toward cervical oncology. Emerging protocols, such as the Rehabilitation After Cervical Cancer trial (NCT06786091), are actively investigating how structured, personalized physical exercise can mitigate treatment-related sequelae, improve pelvic floor function, and rescue systemic immune competence in cervical cancer survivors post-chemoradiation. Crucially, it must be emphasized that the clinical benefits of exercise oncology, such as improved quality of life, reduced cancer-related fatigue, enhanced treatment tolerance, and improved functional capacity, are already firmly established and clinically validated. These patient-centered benefits stand independently of whether the underlying molecular and myokine-mediated mechanisms are fully characterized or validated. While uncovering the myokine axis is scientifically paramount to refine future protocols, the clinical prescription of exercise does not need to wait for mechanistic validation to justify its implementation in standard oncological care.

To transition from generic recommendations to true “precision exercise” protocols in gynecological oncology, must be dosed with the same rigor as pharmacological agents, detailing type, intensity, volume, and frequency of the exercise. Current evidence indicates that different exercise modalities yield distinct adaptations. For instance, to counteract the hyperinsulinemia and visceral adiposity driving endometrial carcinogenesis, concurrent training programs combining moderate-intensity continuous aerobic exercise (MICT: 150 minutes/week at 50–70% heart rate reserve) with progressive resistance training are highly effective, as they chronically optimize insulin sensitivity and sustain continuous baseline FGF21 and Irisin circulation. In contrast, in the advanced ovarian cancer setting, where suppressing tumor niche immunosuppression is a priority, high-intensity interval training (HIIT: e.g., 4x4 minute bouts at 85–95% peak heart rate, twice weekly) is hypothesized to be a superior trigger for acute pulsatile IL-6 and IL-15 release, which are essential to transiently drive NK and T-cell infiltration into the tumor microenvironment. Detailing these specific variables (Frequency, Intensity, Time, and Type) across active trial protocols, such as the STRIVE Cardio Program (NCT06534008) focusing on structured aerobic and resistance training, is crucial. Only by customizing these mechanical and metabolic workloads can clinician-scientists systematically exploit the muscle-tumor axis to design targeted, patient-specific complementary oncological therapies.

To maximize the clinical utility of the muscle-tumor axis in gynecological oncology, the translational evidence must be systematically segregated based on the specific clinical window of intervention. The biological endpoints, myokine dynamics, and exercise prescription paradigms differ fundamentally when deployed for primary cancer prevention versus active oncological management or survivorship.

### 6.2 The oncological prevention track: risk reduction and premalignant interventions

In the prevention setting, the clinical goal is primary risk reduction—specifically targeting the systemic, chronic low-grade inflammation, insulin resistance, and altered adipokine-myokine balances that characterize obesity-induced niches. Here, long-term, sustained physical exercise operates as an endogenous prophylactic intervention. Chronic adaptations to aerobic and progressive resistance training sustain elevated baseline levels of protective factors like FGF21 and Irisin, which collectively counteract metabolic hyperinsulinemia and reverse the early hyperplastic signals driving endometrial and ovarian carcinogenesis. In patients with known high-risk premalignant conditions (such as atypical endometrial hyperplasia or severe metabolic syndrome), this regular myokine exposure can potentially slow down or arrest initial cellular transformation before invasive malignancy develops.

### 6.3 The interventional and therapeutic track: active therapy, adjuvant settings, and survivors

When exercise is introduced post-diagnosis, the clinical context shifts to active therapy, adjuvant settings, or survivorship care. During active treatment (e.g., concurrent chemotherapy or pelvic radiation for cervical and ovarian cancers), the primary objective is to maintain functional capacity, mitigate severe treatment-induced toxicities, and prevent muscle wasting (sarcopenia). In this active window, pulsatile myokine spikes induced by personalized acute exercise bouts (such as moderate-intensity continuous exercise or targeted resistance protocols) act as acute immune-modulators, physically driving cytotoxic NK and T-cell homing into the remaining tumor beds and enhancing treatment tolerance. In the adjuvant and survivorship phases (e.g., evaluated in protocols like the STRIVE Cardio Program [NCT06534008] and the Rehabilitation After Cervical Cancer trial [NCT06786091]), the focus transitions toward long-term systemic rehabilitation, preventing cancer recurrence, and reversing the structural and metabolic sequelae of multi-modal oncological care.

Clinical trials are summarized in **Table 2**. The future of the field lies in the development of “precision exercise” protocols (32, 77). By tailoring the modality (aerobic vs. resistance), intensity, and timing of physical activity, it may be possible to maximize the secretion of specific myokines, such as Irisin for metabolic reprogramming or IL-15 for NK-cell-mediated immunosurveillance (32). This approach repositioned skeletal muscle as a primary driver of oncological protection, offering a tailored, non-pharmacological strategy to improve clinical outcomes in obesity-driven gynecological cancers.

**Table 2:** Registered clinical trials of physical activity and exercise interventions in gynecologic oncology.

Study title (short)	NCT ID	Cancer type	Intervention	Setting	Primary outcomes	Status
Exercise + Nutrition During Chemotherapy	NCT05761561	Ovarian, Endometrial	Combined exercise + nutrition program	During treatment	Feasibility, QoL, treatment tolerance	Recruiting
STRIVE Cardio Program	NCT06534008	Endometrial	Supervised aerobic + resistance training (12 weeks)	Survivorship	Cardiovascular fitness, physical function	Not yet recruiting
Home-Based Exercise Intervention	NCT06213571	Endometrial	Remote/home-based exercise	Survivorship	Strength, physical activity levels	Recruiting
Exercise Preconditioning (HIIT)	NCT05029154	Ovarian	High-intensity interval training (HIIT)	Pre-treatment	Surgical outcomes, fitness	Recruiting
Exercise in Ovarian Cancer Survivors	NCT03364673	Ovarian	Structured exercise program	Survivorship	QoL, fatigue, distress	Completed
Physical Activity Intervention Study	NCT03641287	Ovarian	Physical activity promotion	Survivorship	Physical activity, QoL	Completed
Online Personalized Exercise Program	NCT06709534	Gynecologic (mixed)	Digital individualized exercise program	Survivorship	Fatigue, sleep, depression, microbiome	Recruiting
Mobile App Physical Activity Intervention	NCT05743517	Gynecologic (mixed)	App-based PA intervention	During treatment	Physical activity adherence, QoL	Recruiting
Rehabilitation After Cervical Cancer	NCT06786091	Cervical	Exercise-based rehabilitation	Post-treatment	Functional recovery, late effects	Not yet recruiting

## 7 Conclusion

Obesity-driven gynecological malignancies arise within a systemic landscape defined by chronic inflammation, hyperinsulinemia, and adipokine dysregulation. While adipose tissue has traditionally been viewed as the primary driver of a pro-tumorigenic milieu, this review highlights the emerging role of skeletal muscle as a critical endocrine modulator that may contribute to counteracting cancer progression. The “muscle–tumor axis” represents a sophisticated inter-organ communication network where exercise-induced myokines may orchestrate a multi-layered defense, although their efficacy appears to be highly context-dependent. These molecular transducers do not merely counteract metabolic derangements signaling; preclinical data suggest they may possess the potential to modulate the tumor microenvironment by enhancing immune surveillance.

To properly contextualize the current state of research, the evidence supporting the muscle-tumor axis must be stratified across different translational frontiers: a) Preclinical Evidence: Highly robust. Cell cultures and animal models demonstrate clear mechanisms by which myokines (e.g., Irisin, IL-15) can inhibit tumor cell proliferation or enhance immune surveillance. b) Observational Evidence: Moderate but associative. Epidemiological data confirm that higher physical activity and preserved muscle mass correlate with better survival and lower recurrence in gynecological cancer patients. c) Interventional Evidence: Emerging. Clinical trials show that exercise modifies systemic biomarkers, reduces systemic inflammation, and improves body composition.

Ttransitioning from these mechanistic models to validated ‘precision exercise’ protocols requires a highly cautious approach. Given that current human data are mostly associative, robust clinical trials are critically needed to firmly establish causality and rule out confounding factors before myokine modulation can be claimed as a definitive clinical reality in gynecological oncology (10, 39).

In this perspective, physical exercise transcends its role as a lifestyle modification and emerges as a potent, biologically active intervention; however, the transition to ‘precision exercise’ requires a cautious approach, moving beyond a ‘one-size-fits-all’ model toward protocols that account for the unique molecular and metabolic landscape of each patient.

Several limitations of the current literature must be acknowledged. First, to our knowledge, there is lack of randomized controlled trials directly linking exercise protocols to tumor outcomes. Secondly, there is a lack of standardized exercise protocols (mode, intensity, and duration) across clinical trials, making it difficult to determine the optimal ‘dose’ required to trigger oncosuppressive myokine pulses in gynecological oncology. Lastly, the confounding influence of persistent adiposity and myosteatosis on myokine secretome remains partially understood, requiring more longitudinal human data to validate these muscle-derived transducers as reliable clinical biomarkers. The clinical integration of myokine signaling offers a promising avenue for “precision exercise” oncology, where tailored protocols can be designed to maximize oncosuppressive pulses even in the presence of persistent adiposity. Future research must focus on identifying specific myokine-based biomarkers to better monitor treatment response and refine non-pharmacological strategies, ultimately improving the oncological trajectory and quality of life for gynecological cancer survivors.
